# Popularity and Perceptions of Protein Supplementation: A Cross-Sectional Study Among Undergraduate University Students Aged 18 to 25 Years in Riyadh, Saudi Arabia

**DOI:** 10.7759/cureus.75431

**Published:** 2024-12-09

**Authors:** Abeer Almudaihim, Kavita Sudersanadas, Ihssan Abdelrahman, Aseel Alkoblan, Winnie Philip, Prachi Tambur, Shatha Alrabiah

**Affiliations:** 1 College of Applied Medical Sciences, King Saud Bin Abdulaziz University for Health Sciences, Riyadh, SAU; 2 King Abdullah International Medical Research Centre (KAIMRC), Ministry of National Guard Health Affairs, Riyadh, SAU; 3 College of Applied Medical Sciences/Respiratory Therapy, King Saud Bin Abdulaziz University for Health Sciences, Riyadh, SAU

**Keywords:** motives, perceptions, popularity, protein supplements, sources of information, types, young adults

## Abstract

Background and aim: Young adults, particularly those aged 18-25, exhibit varying perceptions and choices regarding the use of protein supplements (PS). Understanding these perceptions can significantly enhance professional guidance and nutrition education for undergraduate students. This study, conducted in Riyadh, Saudi Arabia, aims to explore the perceptions of PS use and identify the most popular PS among university students.

Materials and methods: This cross-sectional descriptive study involved young adults aged 18-25 of both genders. A validated online questionnaire was used to collect data, which was analyzed using IBM SPSS Statistics version 21 (IBM Corp., Armonk, United States). A p-value of <0.05 was considered statistically significant. The Institutional Review Board (IRB) of King Abdullah International Medical Research Centre (KAIMRC) approved the study protocol.

Results: A total of 740 participants (430 females (58.1%) and 310 males (41.9%)) were included in the study. Around 17% reported using PS, with males being the primary users (65 (51.6%)). Protein powder was the most popular protein supplement (48 (38.1%)), followed by a combination of protein powder, protein bars, and fortified foods (42 (33.3%)). Age, gender, and family income significantly influenced protein supplement use (p<0.05). Males preferred a combination of protein powder and fortified food. Significant gender differences were observed in the choice of protein supplement type (p=0.001). Perceptions of PS were influenced by friends, family, doctors, pharmacists, social media, and the internet. However, the majority of participants indicated a need for more awareness regarding PS.

Conclusion: The study underscores the need for improved professional guidance and nutrition education for young adults, particularly in addressing misconceptions and gender differences in protein supplement use.

## Introduction

Protein supplements (PS) are products commonly used for various purposes such as muscle building, weight loss, exercise recovery, and improving endurance and cardio performance [1]. These supplements can significantly impact health and well-being, as evidenced by studies showing improved quality of life, aerobic capacity, body composition, and digestive health [2]. PS are often perceived as essential by athletes, potentially due to misleading claims made by manufacturers [3]. Research indicates that PS can aid muscle recovery after physical stress, reducing muscle soreness and promoting faster muscle regeneration [4]. For people involved in operations with strenuous physical activity and inadequate dietary intake, protein supplementation may be beneficial for maintaining muscle mass and meeting metabolic demands [5]. In addition, research shows that protein supplementation can aid in muscle injury rehabilitation by reducing muscle soreness, promoting faster muscle fiber regeneration, and improving contractile ability after physical exertion [5].

PS usage is a prevalent trend among university students, focusing on young males aiming to increase muscle mass. Research indicates that a significant percentage of male and female students use dietary and muscle-enhancing dietary supplements for various reasons, such as improving physical or mental performance, increasing muscle mass, and enhancing appearance [6]. However, it is noteworthy that male students [7], in general, are more likely to use dietary supplements, with PS being one of the most commonly used types. The prevalence of PS consumption has increased among adult students [8] in the Middle East [9] and Asia [10].

These supplements can be drinking mixes containing base protein powder and other active ingredients tailored to specific demographics or activities [11]. In Saudi Arabia, PS are popular among athletes and non-athletes. However, there is a lack of awareness and harmful practices associated with their use, impacting renal function and lipid profiles [12].

Protein is one of the most essential nutrients; it significantly increases muscle mass and fat loss. While PS may have many beneficial effects, such as lowering blood pressure, antioxidant defense mechanisms, decreased incidence of breast cancers, and reduced severity of menopausal symptoms, specifically for women, they also have a few adverse effects on bone health, metabolism, hepatic, and renal function if not taken in the correct dose or frequency [13]. There are numerous sources of dietary protein, including regularly ingested food products such as eggs, animal meat, dairy products, and vegetables (natural protein sources), as well as PS that come in the form of powders (animal-based protein powders are casein, whey, and collagen), pills, premade shakes, bars, or other foodstuffs [14]. 

The recommended dietary allowance (RDA) for adult men and women is 0.8 g of protein per kg of body weight per day. The recommendations for physically active individuals are slightly higher than the public. The recommendation for protein for endurance athletes and muscle building is 1.2 to 1.7 g/kg of body weight per day. However, all recommendations range below 2 g/kg daily [15]. Earlier studies reported that college male athletes have the perception that their RDA is more than twice the RDA (0.8 g/kg) and often >2 g/kg [16]. 

In Saudi Arabia, a survey from Riyadh indicated that most young adult males used dietary supplements, and the most consumed supplements were proteins. In addition, the popularity of PS is higher among males than females [17].

Many studies on nutrition information sources showed that individuals often use dietary supplements without scientific evidence - only a tiny proportion reported seeking professional advice from a dietician [18]. However, most users rely mainly on the Internet, pharmacists, and sports coaches [19].

There is increased usage of PS among university students, particularly in the context of improving health, fitness, and academic performance. Protein supplementation is widely discussed in health and fitness literature, and its popularity has been growing globally, including in Saudi Arabia. Hence, the study was conducted with a specific aim to evaluate the popularity and perceptions of PS usage among university students in Saudi Arabia.

The study results will help to identify the perspectives of PS usage and shed light on their dietary choices, particularly regarding the types and patterns of supplement consumption. This information can be utilized to inform future nutrition interventions, health campaigns, and policies promoting balanced nutrition.

## Materials and methods

Study participants

The study participants were undergraduate university students of both genders, aged 18-25, of any nationality. Inclusion criteria included age between 18 and 25 and regular studentship in a university located in Riyadh, Saudi Arabia. To participate in the study, the concerned person must understand and provide written informed consent.

Study design

The study was designed using a cross-sectional descriptive study design.

Sample size

The sample size was calculated using the Raosoft online calculator (Raosoft Inc., Seattle, United States), a software program recommended for population surveys, to meet the desired confidence level of 95% and margin of error, usually 5% [20]. The total population of the selected universities of undergraduate students in Riyadh is approximately 209,746, according to the Higher Education Statistics Centre of the Ministry of Education [21]. Therefore, to achieve a confidence level of 95% and a margin of error of 5% [22], a minimum sample size of 384 was required as an adequate sample size. However, assuming a 50% non-response rate, the final sample size was calculated using the following formula:

Final sample size = Effective sample size / (1 - non-response rate anticipated) [23].

Final sample size = 384 / (1 - 0.5) = 768, rounded off to 770.

Sampling technique

The study used convenience sampling, a non-probability sampling method that relies on collecting data from people who are convenient and available to participate.

Data collection and management

The data was collected from September 3, 2019, to November 30, 2019. The research team developed a validated self-administered questionnaire (see in Appendices) with open and closed-ended questions to collect the data. The questionnaire contained questions related to demography, perception, usage of PS, and consumption patterns. It was translated from English into Arabic following the guidelines for translating a questionnaire. In addition, the respondents' knowledge of the recommended dietary allowances (1 g/kg of body weight) [24] was identified by asking them about the exact amount of protein they require daily. Furthermore, the knowledge about the regulatory authority involved with the safety of PS was acquired by querying whether they know the PS in the market and whether they are tested by competent authorities (Saudi Food and Drug Administration) [25].

A pilot test was conducted among 30 eligible students, whose data were subsequently excluded from the study while the analysis was being done. Cronbach's α, a reliability estimate, was applied to the questionnaire to assess its internal consistency. The questionnaire scored 0.723, which indicated that the items were consistent, making the measure reliable.

The questionnaire was translated from English into Arabic following the guidelines for translating a questionnaire. The questionnaires were distributed in English and Arabic as Google forms through the participants' official emails from the concerned universities in Riyadh.

Descriptive analysis was performed for categorical variables, presenting frequency and percentage. Shapiro-Wilk test was conducted to check the normality of the data. The association between gender and perceptions toward PS use and the demographic variables associated with PS use were assessed using the Pearson chi-square and Fisher's exact tests. A p-value of less than 0.05 was considered statistically significant. All statistical analyses were conducted using IBM SPSS Statistics version 21 (IBM Corp., Armonk, United States).

Ethical approval

The study protocol was approved (approval memo reference IRBC/1424/19 dated 21-08-2019) by the Institutional Review Board (IRB) of King Abdullah International Medical Research Centre (KAIMRC), Ministry of National Guard Health Affairs (MNGHA), Riyadh, Saudi Arabia.

## Results

The participants' response rate was 96.10%, and 740 college students from six universities in Riyadh participated in the study.

Baseline characteristics of the respondents and their influence on PS usage

The baseline characteristics of the individuals and their influence on PS usage are presented in Table 1. 

**Table 1:** Baseline characteristics of the respondents and their influence on PS usage (N=740) * Statistically significant at 5%; ** Values are presented as frequency (percentage); ^a^ Pearson chi-square test PS: Protein supplements

Characteristics	Total**	PS users (n_1_=126)	PS non-users (n_2_=614)	^a ^p-value
Nationality				
Saudi	642(86.8)	110(87.3)	532(86.6)	
Non-Saudi	98(13.2)	16(12.7)	82(13.4)	0.843
Age in years				
18-21	345(46.6)	49(38.9)	296(48.2)	
21-24	326(44.1)	59(46.8)	267(43.5)	0.045*
>24	69(9.3)	18(14.3)	51(8.3)	
Gender				
Males	310(41.9)	65(51.6)	245(39.9)	0.015*
Females	430(58.1)	61(48.4)	369(60.1)	
Marital status				
Single	711(96.1)	119(94.4)	592(96.4)	0.312
Married	29(3.9)	7(5.6)	22(3.6)	
Type of residence				
Unaccompanied	62(8.4)	10(7.9)	52(8.5)	0.844
Family accommodation	678(91.6)	116(92.1)	562(91.5)	
Type of university				
Government	523(70.7)	93(73.8)	430(70)	0.396
Private	217(29.3)	33(26.2)	184(30)	
Monthly income (USD)				
≤250	510(68.9)	68(54)	442(72)	0.001*
250-1000	164(22.2)	43(34.1)	121(19.7)	
>1000	66(8.9)	15(11.9)	51(8.3)	
Total	740(100)	126(100)	614(100)	

The majority of the respondents were Saudi nationals (86.80%), including 430 females and 310 males aged 18-25. Age, gender, and family income were the significant factors influencing PS usage (p=0.045, 0.015, and 0.001, respectively). 

Table 2 shows the monthly expenditures of PS users based on gender.

**Table 2:** Gender-based monthly expenditure for PS among the PS users (n1=126) * A p-value of less than 0.05 was considered statistically significant; ** Values are presented as frequency (percentage); ^a^ Pearson chi-square test PS: Protein supplements

Monthly expenditure (in USD) on PS	Females**	Males**	Total**	p-value*
<25	23(38.3)	21(31.8)	44(34.92)	
25-100	32(53.3)	37(56.1)	69(54.8)	0.650 ^a^
100-250	5(8.3)	8(12.1)	13(10.3)	
Total	60(47.61)	66(52.38)	126(100)	

The respondents who used PS spent around 25-250 USD monthly to purchase it. No statistically significant difference (p=0.650) was found between gender and PS expenditure.

Influence of nutritional status of the respondents on PS usage

Table 3 details the nutritional status of the respondents based on body mass index (BMI). The respondents had a median BMI of 23 kg/m^2^.

**Table 3:** Nutritional Status of the respondents (N=740) * Numbers in parenthesis indicate percentage PS: Protein supplements; BMI: Body mass index

BMI categorization	Type of PS usage*		Total (N=740)
	Users (n_1_=126)	Non-users (n_2_=614)	
Underweight	10(7.9)	55(9)	65(8.8)
Normal	66(52.4)	335(54.6)	401(54.2)
Overweight	37(29.4)	132(21.5)	169(22.8)
Obese	13((10.3)	92(15)	105(14.2)
Total	126(100)	614(100)	740(100)

About 52.4% of the respondents were normal, whereas 7.9% were underweight, and 39.7% were either overweight or obese. Chi-square analysis indicated that nutritional status did not influence PS usage (χ2 = 4.653, p=0.199).

Preference for various types of PS by the users

Protein bars (PB), protein powder (PP), and fortified food with protein (FFP), either alone or in combination, were found to be consumed by the PS users. The gender-wise preference of various types of PS for consumption by the respondents is given in Table 4.

**Table 4:** Gender wise preference of different PS (n1 =126) ^a^ Numbers in parenthesis indicate a percentage; ^b^ Fisher's exact test; ^c^ Pearson chi-square test; * Statistically significant at 5% PS: Protein supplements

Type of PS	Total	Gender-wise preference rates ^a^ for PS type		p-value
		Males	Females	
Protein bar ^c^	12(9.5)	5(41.7)	7(58.3)	0.552
Protein powder ^c^	48(38.1)	31(64.6)	17(35.4)	0.022*
Fortified food with protein ^b^	4(3.2)	3(75)	1(25)	0.620
Protein bar and protein powder ^b^	6(4.8)	3(50)	3(50)	0.629
Protein bar and fortified food with protein ^b^	4(3.2)	0	4(100)	0.052
Protein powder, protein bar, and fortified food with protein ^c^	42(33.3)	13(31)	29(69)	0.001*
Protein powder and fortified food with protein ^b^	10(7.9)	10(100)	0	0.001*
Total	126(100)	65(51.59)	61(48.41)	

PP was the most preferred or popular item among the respondents (38%), followed by a combination of PP, PB, and FFP (33.33%). Gender influences the choice of PP (p=0.022) and the consumption of a combination of PP, PB, and FFP (p=0.001) as well as PP and FFP (p=0.001). Males preferred PP (64%) and a combination of PP and FFP (100%), whereas females preferred a combination of PP, PB, and FFP (69%).

Consumption pattern of PS and the motives to use PS

Most PS users (68.73%) consumed PS daily, 18.17% consumed PS weekly, and 12.64% consumed PS once a month. Table 5 details the consumption pattern of PS by the respondents.

**Table 5:** Consumption pattern of PS (n1=126) * Numbers in parenthesis indicate percentage PS: Protein supplements

Consumption pattern	Number of respondents*
Daily use of PS	
Once	69(54.51)
Twice	15(11.85)
More than twice daily	3(2.37)
Total	87(68.73)
Weekly use of PS	
Once	6(4.74)
Twice	7(5.53)
More than twice weekly	10(7.90)
Total	23(18.17)
Monthly use of PS	
Once in a month	16(12.64)
Total	16(12.64)
Grand total	126(100)

**Figure 1:**
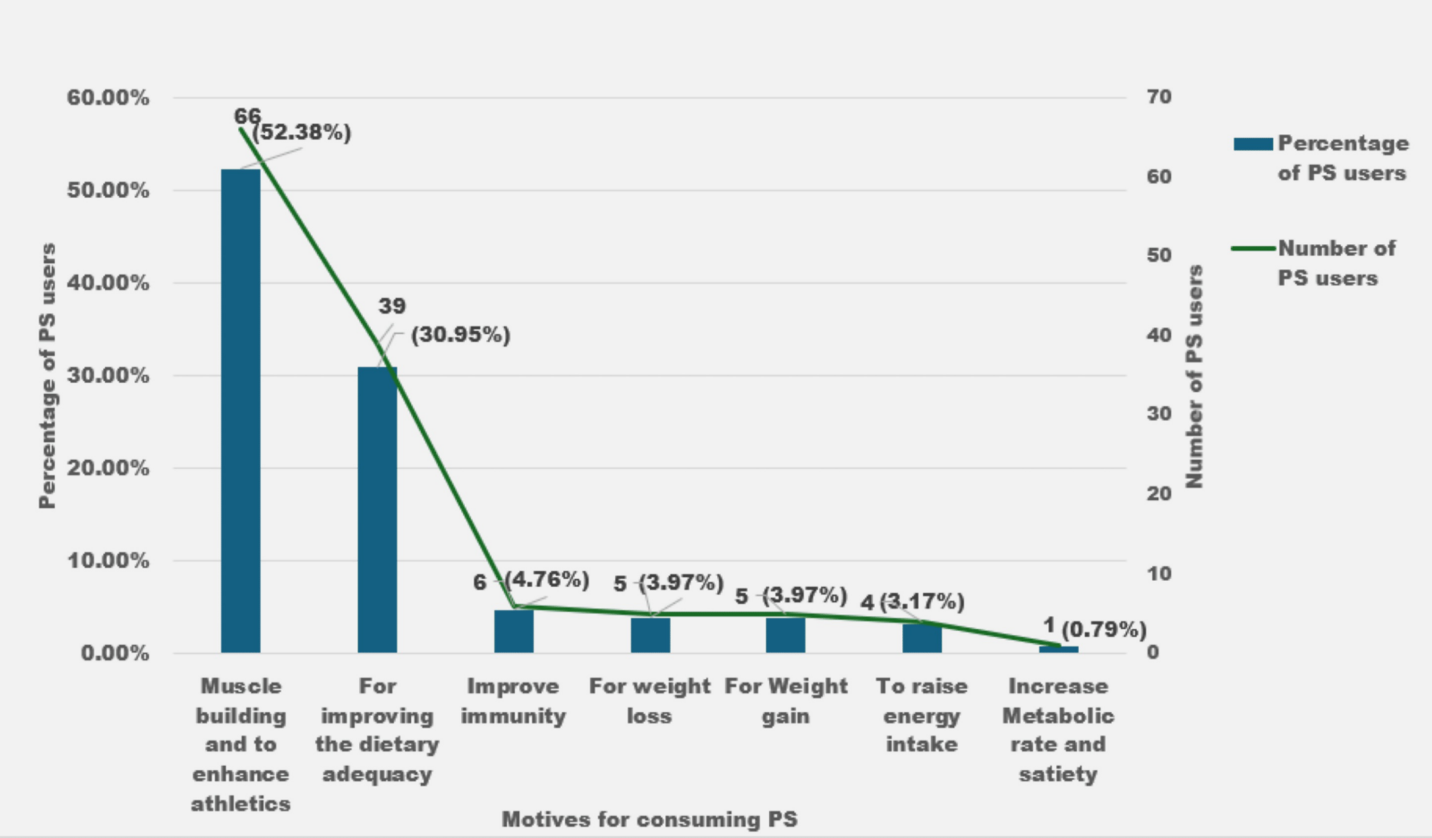
Motives for the consumption of PS PS: Protein supplements

The various motives for using PS (n_1_=126) are given in Figure 1.

Most subjects (52.38%) consumed PS to build muscle, enhance athletics, and improve dietary adequacy (30.95%). The internet (44.32%), social media (36.49%), doctors and pharmacists (24.14%), gym trainers (18.92%), and salespeople of PS shops (11.21%) formed major sources of information for the PS users.

Internet-based sources of information influenced the regular consumption of PS (p=0.005). Friends and family (p=0.028) and social media (p=0.012) influenced the development of the motive to consume PS, which can improve immunity; hence, PS consumption was believed to enhance public health at large.

Perceptions of the respondents about PS usage

The different perceptions of the respondents are presented in Table 6.

**Table 6:** Perceptions of the respondents about PS usage (N=740) * Statistically significant at 5%; ^a^ Fisher's exact test; ^b^ Pearson chi-square test PS: Protein supplements

Particulars	Type of use of PS*			
	PS users (n_1_=126)	PS non-users (n_2_=614)	Total (N=740)	p-value
Perception of usage of PS ^a^				
To get a cure for an illness	2(1.6)	66(10.7)	68(9.2)	
To enhance athletics	40(31.7)	211(34.4)	251(33.9)	
To add the quality for vegetarian diet	0	14(2.3)	14(2.3)	
Anyone can use PS as a healthy food	49(38.9)	155(25.2)	204(27.6)	
Subjects with multiple perceptions	35(27.78)	168(27.36)	203(27.43)	0.001*
Knowledge about recommended dietary intake of protein ^b^	79(62.69)	66(10.75)	145(19.6)	0.001*
The belief that PS are healthy ^b^	108(85.7)	316(51.5)	424(57.3)	0.001*
Knowledge about existence of regulatory authorities of PS for food safety such as Saudi Food and Drug Authority ^b^	93(73.8)	321(52.3)	414(55.9)	0.001*
Will motivate others to consume PS ^b^	91(72.2)	195 (31.8)	286(38.6)	0.001*
Practice of reading the nutrition labels of PS before consumption ^a^	122(96.82)	590(96.09)	712(96.2)	0.467

There was a statistically significant difference between perceptions of PS users and non-users concerning PS consumption, knowledge of recommended dietary intake (RDI) of protein, PS as a healthy food, and knowledge about regulatory authorities of PS (p=0.001). In addition, 72% of the PS users reported that they would motivate others to consume PS (p= 0.001). The PS users and non-users (96%) commonly believed that before consuming PS, it is necessary to read the nutrition labels (p=0.467).

Most (81.4%) of the study population was unaware of the RDI of protein, while 62.69% of the PS users were aware of the RDI. The knowledge of RDI of protein was highly influenced by information from friends and family, doctors and pharmacists (p=0.001), social media (p=0.022), and the internet (p=0.001). Friends and family influenced young adults' beliefs that PS is healthy (p=0.010). However, social media influenced them to read the nutrition labels before consuming PS (p=0.037).

## Discussion

The study included 740 college students from six universities in Riyadh, achieving a response rate of 96.10%. Key findings indicated that age, gender, and family income significantly influenced PS usage. Most respondents were Saudi nationals aged 18-25, with daily consumption at 68.73%. The primary reasons for PS usage included muscle building and dietary enhancement, with the internet being the primary information source. Notably, 81.4% of participants were unaware of their RDI of protein, despite a significant portion of PS users being informed through friends, family, and healthcare professionals.

It was observed that PS was popular among 17% of the study population of undergraduate students. A similar percent popularity rate of 17% was also reported from the United States [5], while that of Indian students was 20.8% [21]. On the contrary, studies from Jordan [9], Lebanon [23], Beirut, and the United States reported a high rate of PS usage of 60.9%, 62%, and 68%, respectively. The earlier studies pointed out that geography and area of residence influenced PS usage [26,27]. However, the type of residence and type of university did not influence the PS usage of the subjects of the present study.

PS usage was significantly influenced by age, gender, and family income. Male students consumed more PS than female students, about 52% and 48%, respectively. This result is concurrent with earlier studies [11,21,27]. Men commonly use PS for bodybuilding and resistance training. The high rate of PS usage among males may be attributed to the fact that males exercised more frequently than females [28].

Family income plays a significant role in influencing college students' use of PS. Research indicates that students from families with a relatively high socioeconomic status are more likely to use healthy, functional foods, including PS [21]. This suggests that students from higher-income families may have better access to and afford PS, leading to increased usage [27]. Moreover, a study in an urban medical college reported that the mean age of PS consumers was around 20.8 years, indicating a trend toward younger individuals using such supplements [11].

The study observed that the most popular PS was PP and a combination of PP, PB, and FFP. There was a gender difference in the choice of PS. Male respondents preferred PP and a combination of PP and FFP, while female respondents preferred a combination of PP, PB, and FFP. The study results are on par with earlier studies [29].

Internet, social media, and friends and family of the respondents influence the regularity of consumption and the development of motives for the consumption of PS. Most of the study participants (81.4%) lacked awareness of the RDI for protein, whereas 62.69% of PS users were familiar with it. An earlier study from Saudi Arabia reported that undergraduate students' nutrition knowledge was less [30]. Understanding of the RDI for protein was significantly influenced by information from friends and family, doctors and pharmacists (p=0.001), social media (p=0.022), and the internet (p=0.001). Influence from friends and family significantly impacted young adults' perception of the healthiness of PS (p=0.010). Furthermore, social media encouraged them to review nutrition labels before consuming PS (p=0.037).

The internet influenced the regularity of PS consumption among college students. Internet-based sources of information significantly influenced the regularity of PS consumption (p=0.005). The reliance on online sources for information on supplements, coupled with the ease of access and the vast amount of information available, significantly impacts the decision-making process of college students when it comes to consuming PS for various purposes, including health benefits, muscle-building, and weight loss [21].

Limitations of the study

The study was conducted among students from universities located in one of the provinces of Saudi Arabia, Riyadh, the capital city. Hence, data related to the influence on the area of residence and PS use was underrepresented.

## Conclusions

The study was conducted among university students aged 18-25 of both genders. They spend 25-250 USD for PS per month. The study highlights the impact of family income and gender on the consumption patterns of PS among college students. The consumption of PS does not influence the nutritional status of the study respondents. PP was the preferred PS, followed by a combination of PP, PB, and FFP. Males preferred PP and a blend of PP with FFP. More than half of the respondents consumed PS for muscle building and a lower percentage for dietary adequacy. The study population's perceptions about PS were based on information from friends and family, doctors, and pharmacists. Online sources such as social media and the internet played a crucial role in decision-making for PS consumption among students. However, social media encouraged reading nutrition labels before the consumption of PS. The majority lacked awareness of protein RDI as social influences shaped perceptions of PS healthiness. The majority of PS users would recommend their peers for PS usage. The study results emphasize the need for targeted nutritional education for students from diverse socioeconomic backgrounds.

## Appendices

**Table 7:** Questionnaire to assess perceptions of protein supplementation among university students # Question number

#	PART I. Demography of the respondents	
1	Name of university	
2	Name of college	
3	Specialization	
4	Age (years)	18-20
		21-23
		24-26
5	Sex	Male
		Female
6	Nationality	Saudi
		Non-Saudi
7	Marital status	Single
		Married
		Widowed/widower
8	Residential status	Living with family
		Independent
9	Family income (Saudi Riyals)	<1000
		1000-3000
		3000-5000
		>5000
10	What is your height?	____ cm
11	What is your weight?	____ kg
12	Body mass index (BMI)	____ kg/m^2^
	PART II. Perceptions of protein supplementation	
13	Do you know how much protein you need daily?	Yes
		No
14	If your answer is “yes”, what is your daily requirement for protein?	____ g
15	Do you think protein supplements are beneficial for your health?	Yes
		No
16	In your opinion, what is the effect of protein Supplements on Health?	Continuous use will protect one from diseases
		Continuous use is harmful to health
		It will not harm health
		It is of no use
		Others
17	Did you know that protein supplements available in the market are tested by a competent authority such as the Saudi Food and Drug Authority?	Yes
		No
18	Do you encourage the use of protein supplements?	Yes
		No
19	In your opinion, for whom can protein supplements be suggested?	Patients
		Athletes
		Vegetarians
		Anyone
20	What is your primary source of information related to protein supplements?	Family and friends
		Doctors and pharmacists
		Social media sites
		Internet
		Protein supplement store employees
		Others
21	Do you think it is necessary to read the ingredients and labels on the packets of protein supplements before consuming them?	Yes
		No
22	Why do you use Protein Supplements? (Choose all applicable)	For muscle building
		Make the vegetarian diet adequate
		To treat illness
		For weight loss
		For weight gain
		To increase athletic performance
		As an energy source
		Others (specify)
23	Do you use Protein Supplements?	Yes
		No
	If your answer is “No,” you have completed the survey. Thank you for your participation	
	PART III. Consumption pattern of protein supplements of protein supplement users	
24	What is the frequency of protein supplement consumption?	Daily once
		Daily twice
		Daily more than two times
		Weekly once
		Weekly twice
		Weekly more than two times
		Monthly once
		Monthly once or twice
		Occasionally
25	What type of protein supplement do you use? (Choose all applicable)	Protein bars
		Protein powder
		Protein fortified foods
		Any other (specify)
26	How many protein supplements do you use at a time?	1 protein bar
		2 protein bars
		More than 2 protein bars
		1 tablespoon of protein powder
		2 tablespoons of protein powder
		More than 2 tablespoons of protein powder
		1 serving of protein fortified foods
		2 servings of protein fortified foods
		More than two servings of protein fortified foods
		Any other (specify)
27	How much do protein supplements cost you per month (Saudi Riyals)?	100-500
		600-1000
		1000-1500
		>1500
	You have completed the survey. Thank you for your participation	
